# 

**Published:** 1917-07

**Authors:** 


### TABLE OF CONTENTS.

The Diagnosis of Acute Osteomyelitis ........ 97

Syphilis of the Testis ........ 106

The Relation of the Laboratory to the Practice of Medicine ........ 114

A Hundred Years Before He is Born ........ 119

Medical Treatment of Gastric Ulcer ........ 124

Medical Items ........ 126

